# Different pattern of stool and plasma gastrointestinal damage biomarkers during primary and chronic HIV infection

**DOI:** 10.1371/journal.pone.0218000

**Published:** 2019-06-11

**Authors:** Lucía Pastor, Jost Langhorst, Dorit Schröder, Aina Casellas, Andreas Ruffer, Jorge Carrillo, Victor Urrea, Sergio Massora, Inacio Mandomando, Julià Blanco, Denise Naniche

**Affiliations:** 1 ISGlobal, Barcelona Institute for Global Health, Hospital Clínic—Universitat de Barcelona, Barcelona, Spain; 2 AIDS Research Institute-IrsiCaixa, Hospital Germans Trias i Pujol, Badalona, Spain; 3 Institut Germans Trias i Pujol (IGTP), Hospital Germans Trias i Pujol, Universitat Autonoma de Barcelona, Badalona, Spain; 4 Centro de Investigação em Saúde da Manhiça (CISM), Maputo, Mozambique; 5 Department of Integrative Gastroenterology, Kliniken Essen-Mitte, Faculty of Medicine, University of Duisburg-Essen, Essen, Germany; 6 Department of Internal and Integrative Medicine, Kliniken Essen-Mitte, Faculty of Medicine, University of Duisburg-Essen, Essen, Essen, Germany; 7 Universitat de Vic—Universitat Central de Catalunya, Vic, Barcelona, Spain; Emory University School of Medicine, UNITED STATES

## Abstract

**Introduction:**

Primary HIV infection (PHI) is the initial phase after HIV acquisition characterized by high viral replication, massive inflammatory response and irreversible immune-damage, particularly at the gastrointestinal level. In this study we aimed to characterize the dynamics of gastrointestinal damage biomarkers during the different phases of HIV infection and assess their association with HIV-disease markers and their accuracy to differentiate PHI from chronic HIV infection (CHI).

**Methods:**

PHI-individuals (n = 57) were identified as HIV-seronegative/HIV-RNA positive and were followed up for one year at the Manhiça District Hospital in Mozambique. Ten plasma and 12 stool biomarkers were quantified by Luminex or ELISA and levels were compared to CHI-naive (n = 26), CHI on antiretroviral-treatment (ART; n = 30) and HIV-uninfected individuals (n = 58). Regression models adjusted by time point were used to estimate the association of the biomarkers with HIV-disease markers. Receiver operating curves were compared for the best accuracy to distinguish PHI from CHI.

**Results:**

Soluble (s)CD14 was significantly associated with the CD4/CD8 ratio (P < 0.05) and viremia levels (P < 0.0001) during PHI. Plasma zonulin and stool lactoferrin were significantly higher in PHI as compared to CHI-individuals (P < 0.05). Plasma zonulin demonstrated the best accuracy to identify PHI among HIV-infected individuals (AUC = 0.85 [95% CI 0.75–0.94]). Using a cutoff value of plasma zonulin ≥ 8.75 ng/mL the model identified PHI with 87.7% sensitivity (95% CI 76.3–94.9) and 69.2% specificity (95% CI 48.2–85.7). An adjusted multivariate model including age, plasma zonulin and sCD14 further increased the classification performance (AUC = 0.92 [95% CI 0.86–0.99]).

**Conclusions:**

While the stool biomarkers did not provide any predictive ability to distinguish PHI from CHI-individuals, plasma sCD14 and zonulin were significantly associated with HIV-disease markers and PHI identification, respectively. These inflammatory biomarkers may be useful to monitor changes in gastrointestinal integrity during HIV infection.

## Introduction

The initial phase after human immunodeficiency virus (HIV) acquisition, usually referred as primary HIV infection (PHI), is characterized by high virus replication and a progressive appearance of HIV-specific antibodies that define seroconversion [1,2]. During PHI there is a massive inflammatory response, an irreversible immune-damage and commonly, a transient febrile illness [3–6]. At this early stage, HIV induces a large depletion of mucosal CD4 memory T cells, particularly in the gut-associated lymphoid tissue (GALT) [7,8]. This depletion is accompanied by a mucosal inflammation and a deregulation of the epithelial barrier maintenance and digestive/metabolic functions [9], resulting in an enteropathy that continues throughout the entire disease course in the absence of antiretroviral-treatment (ART) [7].

Inflammatory Bowel Diseases (IBD) such as Crohn’s and Ulcerative colitis also severely impact the GALT. Many clinical and pathological manifestations of IBD are absent from HIV such as ulceration, colon friability or bleeding. However, both diseases lead to GI tract abnormalities such as immune activation, inflammation, histological-damage including villous atrophy and crypt hyperplasia, decreased absorption, increased intestinal permeability and microbial imbalance [9–17]. Many of these gut specific changes have translated into the identification of diagnostic and prognosis biomarkers for active IBD, including biomarkers of neutrophil and eosinophil activation, innate immunity and intestinal permeability [18–21]. Although plasma biomarkers [1,22–25] and stool microbiota [26,27] have been widely described in HIV-infected patients; to our knowledge, stool biomarkers of GI inflammation and damage have not been characterized during the different phases of HIV-infection.

Retaining GALT integrity and maintaining low immune activation has been suggested to protect the host from continued CD4 T-cell depletion and progression to AIDS [1,28–30]. Thus, understanding the dynamics of GI-damage biomarkers during PHI may reveal specific changes in expression patterns of biomarkers which can be used as indicators of diseases progression at an early stage. In addition, since these biomarkers might differ between PHI and chronic HIV infection (CHI), we hypothesized that one or more might serve as an incidence biomarker. Indeed, accurate cross sectional estimates of HIV incidence of new infections are critical for planning and evaluating the success of HIV interventions. In 2011 the WHO/UNAIDS Incidence Assay Critical Path Working Group set criteria for discovery of new biomarkers that could improve upon the existing cross sectional serological-based HIV incidence assay, which is dependent on antibody titers found to be severely impaired after ART treatment [31].

This study thus sought to characterize the kinetics of minimally-invasive inflammation biomarkers during the first year of HIV infection, assess their association with HIV-disease markers and determine their predictive capacity as incidence biomarkers.

## Materials and methods

### Ethics statement

This study was approved by local institutional review boards at Barcelona Clinic Hospital (2011/6264) and by the Ministry of Health of Mozambique (461/CNBS/12). All methods were carried out in accordance with the relevant guidelines and regulations. Written informed consent was obtained from all subjects (both HIV-infected and HIV-uninfected subjects) prior to participation.

### Study population

The study population was enrolled between April 2013 and November 2014 at the Manhiça District Hospital (MDH) in Manhiça, Southern Mozambique. All study participants were over 18 years old and residents of the District Surveillance System (DSS) study area. The present analysis is a sub-study of a prospective cohort of primary HIV-infected adults enrolled and followed up for 12 months. The current study population is thus a convenience sample, nested in the Gastro-intestinal biomarkers in acute-HIV infected Mozambican adults study (GAMA) [32–34].

### HIV diagnosis and clinical follow up

During the screening, subjects presenting to the outpatient clinic of MDH for non-specific febrile symptoms or voluntary HIV counseling and testing were included in the PHI group if they were negative or indeterminate for rapid test serology and HIV-RNA positive for pooled-viral load (VL) testing, according to the screening profile previously characterized [32]. A control population was established by random selection among HIV-uninfected and individuals were invited to attend a study visit 1 month after the screening date [32]. PHI individuals enrolled were followed up at seven consecutive visits 1, 2, 3, 4, 6, 9 and 12 months after the screening visit and left the study when they reached criteria for ART initiation according to the current national guidelines [32,33]. CHI patients were included in the CHI-naïve (n = 26) or the CHI-ART (n = 30) as previously described [33], if they had previously initiated treatment, mostly based on a zidovudine/lamivudine/nevirapine regimen. Technical information and procedures regarding HIV diagnosis, clinical follow up, microbiological evaluation, HIV-specific antibody determination and biomarker quantification have been described in detail previously [32–34].

### Staging of primary HIV infection and quantification of biomarkers

VL and Western Blot HIV-serology at screening visit were employed to categorize PHI individuals into Fiebig stages as described in previous work [32,33,35]. Visit time points were shifted according to estimated days-post infection by Fiebig stage to approximate similar time since infection during PHI [33,35–37].

Multiplex biomarker profiling was performed for a total of 10 GI-damage biomarkers in plasma samples: interleukin (IL)-17, vascular endothelial growth factor (VEGF), fatty acid-binding protein 2 (FABP2), lipopolysaccharide binding protein (LBP), soluble (s)CD14, zonulin, endogenous endotoxin core antibodies (EndoCAb) IgG, EndoCAb IgM, anti-Saccharomyces cerevisiae antibodies (ASCA) immunoglobulin (Ig)A and IgG; and 12 in stool samples: claudin3, calprotectin, PMN-elastase, zonulin, eosinophil protein X/eosinophil derived neurotoxin (EPX/EDN), stool human ß-Defensin 2 (HBD2), secretory IgA α1–antitrypsin, S100A12, lactoferrin, ASCA and perinuclear antineutrophil cytoplasmic antibodies (pANCA). Determinations in plasma samples were performed in Barcelona laboratories by ELISA commercial assays or Luminex technology as previously described [32–34]. Determinations in stool samples were performed in Essen facilities by ELISA commercial assays according to the manufacturer’s instructions for claudin3, calprotectin, PMN-elastase, zonulin, EDN/EPX, HBD2, secretory IgA, α1–antitrypsin and S100A12 (Immundiagnostik, Bensheim, Germany); lactoferrin, ASCA and pANCA (TechLab, Blacksburg, USA), as regularly performed for IBD diagnosis or prognosis. Overflow and under limit of detection values for every biomarker was standardized as the double and the half of the detection limit, respectively [38].

### Statistical analysis

Proportions were compared using chi-square test. Group comparisons for biomarker levels were performed using the nonparametric Kruskal-Wallis test. Individual comparisons between the different groups were performed using posthoc pairwise comparisons with the Tukey and Kramer (Nemenyi) test. Individual and group comparisons of left-censored data were performed using Peto-Peto test with Holm adjust for multiple comparisons. Spearman’s correlation was used to assess the strength of relationship between zonulin levels in plasma and stool. Differences or correlations were considered significant if P < 0.05.

Relative changes (Z-score) with respect to the HIV-uninfected group were represented by a transformation of the fitted longitudinal models by subtracting the mean and dividing by the standard deviation of HIV-non infected distribution, after a logarithmic transformation in the cases where it was required in order to correct non-normal distributions. Biomarker values, VL and the CD4/CD8 ratio were always log transformed for a better adjustment of skewed data. Mixed-effects models adjusted by visit time point were used to estimate the effect of the biomarker levels on HIV-disease markers, including: the CD4/CD8 ratio, VL and on the report of an intestinal-complaint, defined as diarrhea, vomiting or stomach pain.

The ability of each biomarker's first determination to distinguish between PHI patients and CHI-naïve was assessed via logistic regression models. A multivariate model was constructed using backward step-wise selection and including sex, age, VL and the biomarkers with an inclusion criterion of P < 0.05 in the univariate analysis. Receiver operating curves (ROC) curves from univariate and adjusted-multivariate models were compared for the best prediction [39]. Optimal threshold was determined through maximization of the nearest to (0,1) method. The level of agreement between the classification methods was assessed by the κ-statistic [40].

Data was analyzed using R-3.3.1 software (R Core Team 2016) and Stata Statistical Software: Release 15 (StataCorp 2017. College Station, TX).

## Results

### Characteristics of the study population

Of the 57 PHI patients identified during the screening process [32,33], 40 individuals attended a follow-up 1 month later as previously described [33]. Fifteen-percent of follow up visits lacked a stool sample. There were no significant differences in age or HIV-RNA VL between PHI patients that provided stool sample and those that did not along the study visits. However, there was a significantly higher proportion of females compared to males that provided stool samples along the study visits (91% vs 75% in females and males respectively, P = 0.007). Table 1 summarizes demographic and clinical characteristics of the study population.

**Table 1:** Clinical and demographic characteristics of study population according to HIV-status.

	1st follow-up visit PHI (n = 40)	HIV-uninfected (n = 58)	CHI-naive (n = 26)	CHI-ART (n = 30)	P-value
**Age (years)** [Mean (SD)]	**27.2 (9.2)**	**27.9 (9.5)**	**38.2 (13.4)**	**42.9 (8.8)**	0.0001**
**Gender** [F (%)]	**24 (60.0%)**	**46 (79.3%)**	**19 (73.1%)**	**19 (63.3%)**	**0.162ᵡ**
**Intestinal infection** [n (%)]	**6 (15.0** **%)**	**11 (19.0%)**	**2 (7.7%)**	**3 (10.0%)**	**0.552ᵡ**
**Intestinal complaint last week** [n (%)]	**12 (30.0%)**	**15 (25.9%)**	**4 (15.4%)**	**2 (6.7%)**	**0.067ᵡ**
**Viral Load (RNA Log 10 copies/mL)** [Median (IQR)	**5.00 (4.48–5.43)**	**-**	**4.51 (3.94–4.90)**	**-**	**0.0117** *
**CD4/CD8 ratio** [Median (IQR)]	**0.47 (0.30–0.71)**	**1.64 (1.26–2.32)**	**0.58 (0.41–0.91)**	**0.58 (0.39–0.87)**	0.0001**

Comparisons for proportions were performed by chi2 testᵡ and continuous variables by Mann and Whitney U-test* for the two group comparison and global comparison by Kruskal Wallis test**. SD, standard deviation; F, females; IQR, interquartile range; PHI, primary HIV infection; CHI, chronic HIV infection; CHI-ART, CHI on antiretroviral-treatment. Intestinal infection includes positive result for bacterial, parasite or protozoa testing in stool sample according to the methodology previously described [33].

### Kinetics of the GI biomarkers during the different stages of HIV infection

**Fig 1:**
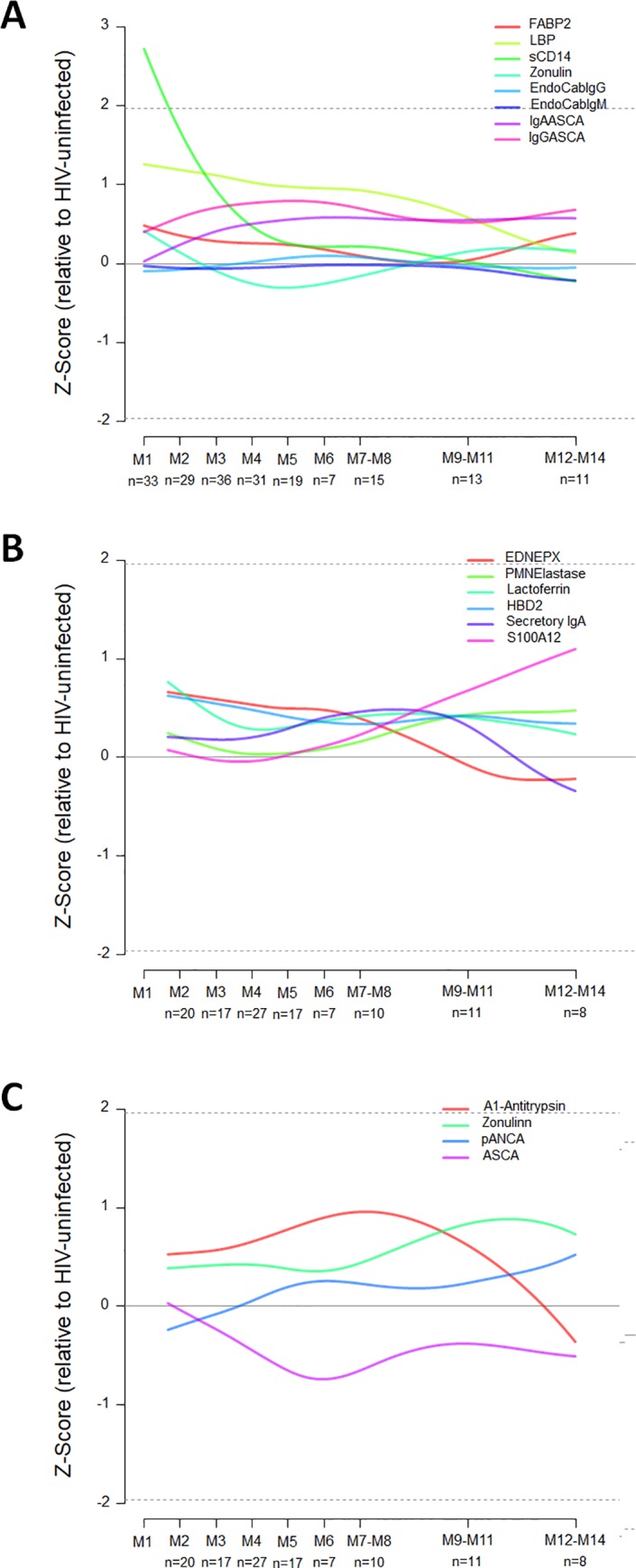
Dynamics of plasma and stool intestinal damage biomarkers during primary HIV-infection (PHI). Dynamics of plasma biomarkers associated with intestinal damage (A) and dynamics of stool biomarkers associated with innate immunity (B) and intestinal permeability (C) are shown. Relative changes (Z-score) of the biomarker levels during PHI with respect to the HIV-uninfected group have been represented by a transformation of the fitted longitudinal models after subtracting the mean and dividing by the standard deviation of HIV-non infected distribution. Values were logarithmic transformed in the cases where it was required to correct non-normal distributions.

Biomarker concentrations by study group are summarized in S1 Table. Biomarker dynamics during PHI were analyzed according to level of expression as well as their relative changes with respect to the levels in the HIV-uninfected group (Fig 1). From the total of 22 biomarkers assessed, plasma VEGF and IL-17 were not quantifiable in more than 75% of the samples and were excluded from the analysis. Levels of stool claudin3 were too low to be comparable between groups and were also excluded from the analysis (S1 Table).

At 2 months post-infection (M2), plasma sCD14 showed the most significant fold difference compared to HIV-uninfected individuals (P < 0.0001, Fig 1A), followed by plasma LBP and ASCA IgG (P = 0.0025 and P = 0.0159, Fig 1A). Stool lactoferrin showed a significant increase at M2 as compared to HIV-uninfected individuals (P = 0.0149, Fig 1B) while there was no significant difference for any of the intestinal permeability stool biomarkers (Fig 1C). Results from the comparison between study groups are summarized in S2 Table.

**Fig 2:**
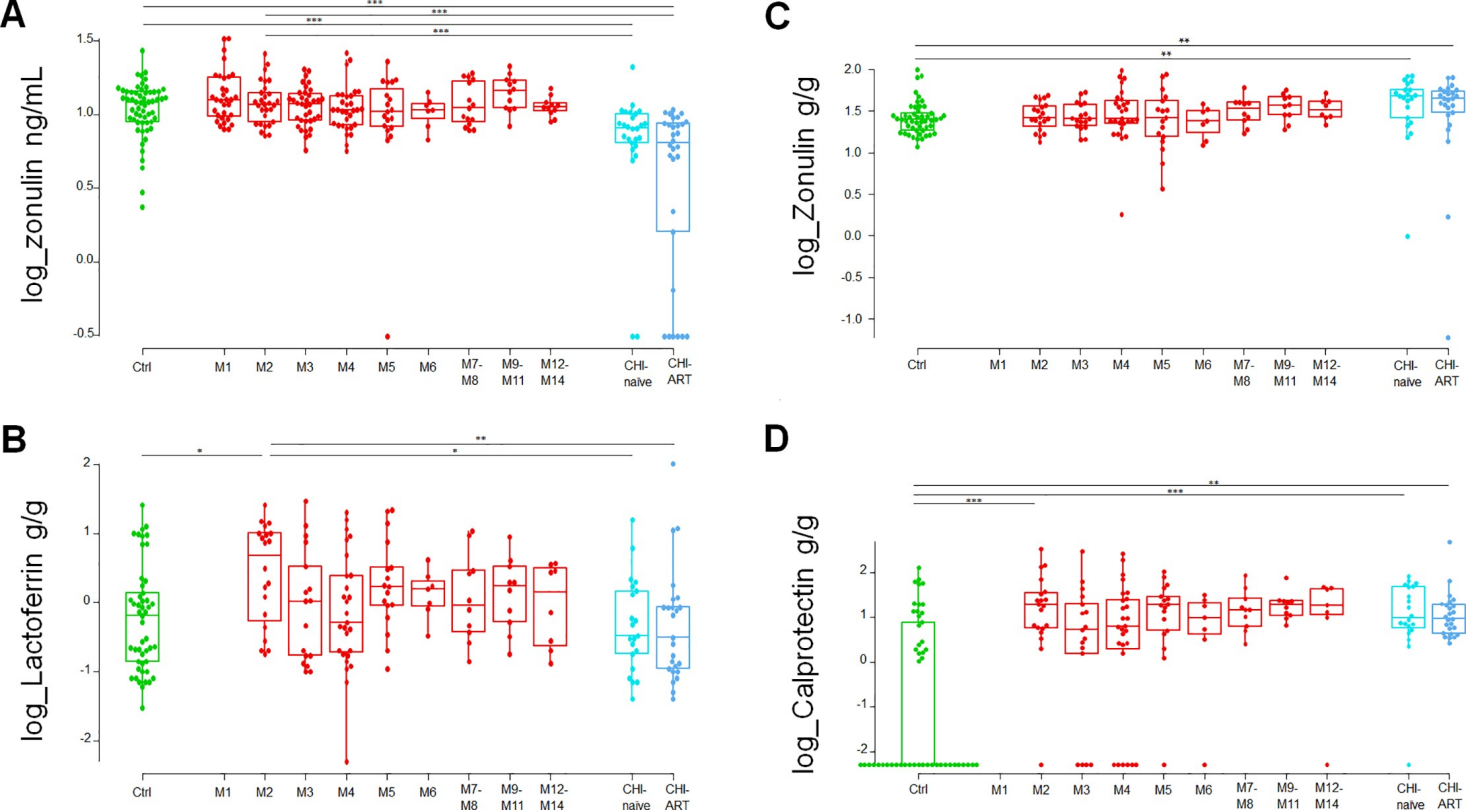
Dynamics of highly significant biomarkers comparing chronic HIV infection (CHI) with PHI and HIV-uninfected individuals (Ctrl). Characterization of plasma zonulin (A), stool lactoferrin (B), stool zonulin (C) and stool calprotectin (D) across different study groups and along time post-HIV-infection. M, months after HIV infection. Box as interquartile range (IQR), middle line as median, whiskers as maximum and minimum, dots as individual observations. Individual comparisons between different groups were performed using posthoc pairwise comparisons with Tukey and Kramer (Nemenyi) test (A, B and C). Individual comparisons of left-censored data were performed using Peto-Peto test with Holm adjust for multiple comparisons (D). Comparisons with PHI group were performed considering the biomarker level at 2-months post-HIV-infection (M2). Significance is indicated as *** if P < 0.001, ** if P < 0.01, and * if P < 0.05.

Comparing biomarker levels at the earliest PHI time point with those in CHI, plasma zonulin was expressed at significantly higher levels at M2 as compared to both CHI-naïve and CHI-ART individuals (P = 0.0006 and P < 0.0001, respectively; Fig 2A) followed by lactoferrin (P = 0.0257 and P = 0.0066, respectively; Fig 2B). Comparing biomarker levels between CHI and HIV-uninfected individuals, plasma sCD14 and stool and plasma zonulin showed significant differences (S2 Table). However, there was no significant correlation between plasma and stool zonulin levels in any of the study groups. Indeed, both CHI groups had significantly higher stool zonulin (Fig 2C) and significantly lower plasma zonulin as compared to HIV-uninfected individuals.

Stool calprotectin was only quantifiable in 45% of the samples of the HIV-uninfected group so its relative change could not be estimated and it was considered left-censored data and analyzed as such as described in methods. All groups of HIV infected individuals showed significantly higher values of stool calprotectin than HIV-uninfected (P < 0.0001), however there were no significant differences in calprotectin levels between PHI and CHI individuals (Fig 2D).

### Association of GI biomarkers with surrogates of HIV disease outcomes

Among PHI or CHI-individuals, there was no significant difference in the median levels of the GI-damage biomarkers between the individuals tested positive for any of the intestinal infection assessed and those that did not.

After evaluation by separate mixed-effect regression models adjusted by time point, sCD14 was the only biomarker showing significant association with the CD4/CD8 ratio or viremia levels during the course of PHI. sCD14 was negatively associated with the CD4/CD8 ratio (P = 0.0402) in PHI-individuals independent of the time point. Each 25% increase in the levels of sCD14 was associated with a 0.83 (95% confidence interval [CI] 0.69–0.99) proportional decrease in the CD4/CD8 ratio. On the other hand, sCD14 was positively associated with VL (P < 0.0001) in PHI-individuals. Each 25% increase in the levels of sCD14 was associated with a 2.12 (95% CI 1.69–2.66) proportional increase in the VL levels.

When assessing correlation with self-reported intestinal complaint, HBD2 was significantly and negatively associated during the first year of HIV-infection (P = 0.0378). A 25% decrease in the levels of stool HBD2 provided an odds ratio of 1.52 (95% CI 1.02–2.27) for self-reported intestinal complaint.

### Evaluation of GI-biomarker accuracy to differentiate primary from chronic HIV infection

**Fig 3:**
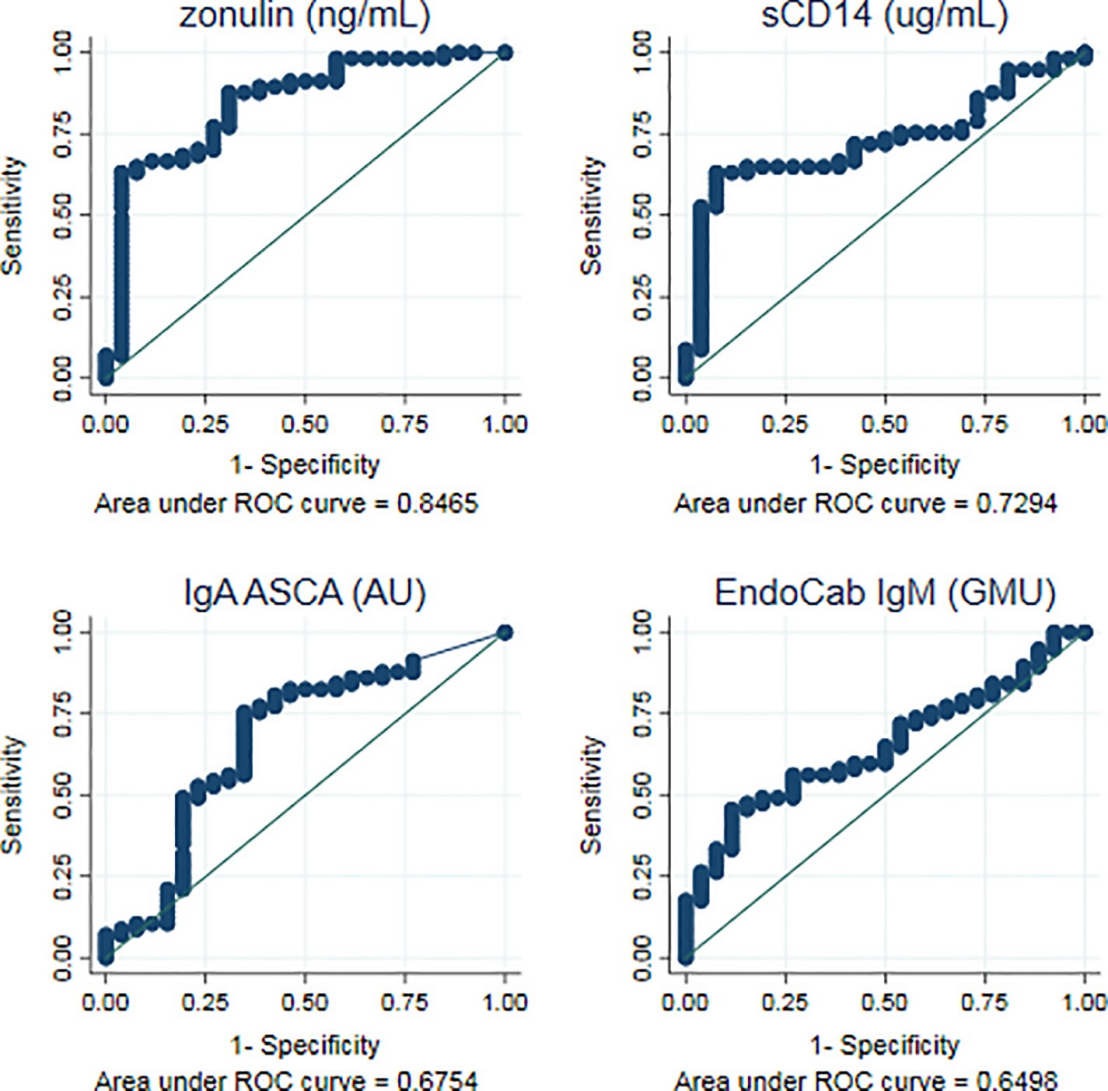
Biomarkers with the best predictive ability to differentiate PHI from CHI-naïve individuals. The ability of each biomarker's first determination to distinguish between PHI patients and CHI-naïve was assessed via logistic regression models. Receiver operating curves (ROC) curves from univariate models were compared for the best prediction. Optimal threshold was determined through maximization of the nearest to (0,1) method. Sensitivity as true positive rate and Specificity as true negative rate. AUC as area under the curve.

In order to assess the capacity of biomarker levels to discriminate between PHI and CHI-naïve individuals, univariate logistic regression models were tested as described in methods. Of the 22 biomarkers tested, plasma zonulin, sCD14, ASCA IgA, EndoCAb IgM and stool secretory IgA showed a significant association with PHI (P < 0.05). According to previous definition [41], ROC analysis of the univariate models from these biomarkers revealed that plasma zonulin had a good classification performance for distinguishing PHI (area under the curve [AUC] = 0.85 {95% CI 0.75–0.94}; Fig 3), while plasma sCD14 showed a fair accuracy (AUC = 0.73 [95% CI 0.62–0.84]), ASCA IgA and endoCAb IgM showed a poor accuracy for PHI identification (AUC = 0.68 [95% CI 0.54–0.81] and AUC = 0.65 [95% CI 0.53–0.77], respectively; Fig 3) and stool secretory Ig A failed to show an acceptable predictive accuracy (AUC = 0.56 [95% CI 0.38–0.74]).

**Fig 4:**
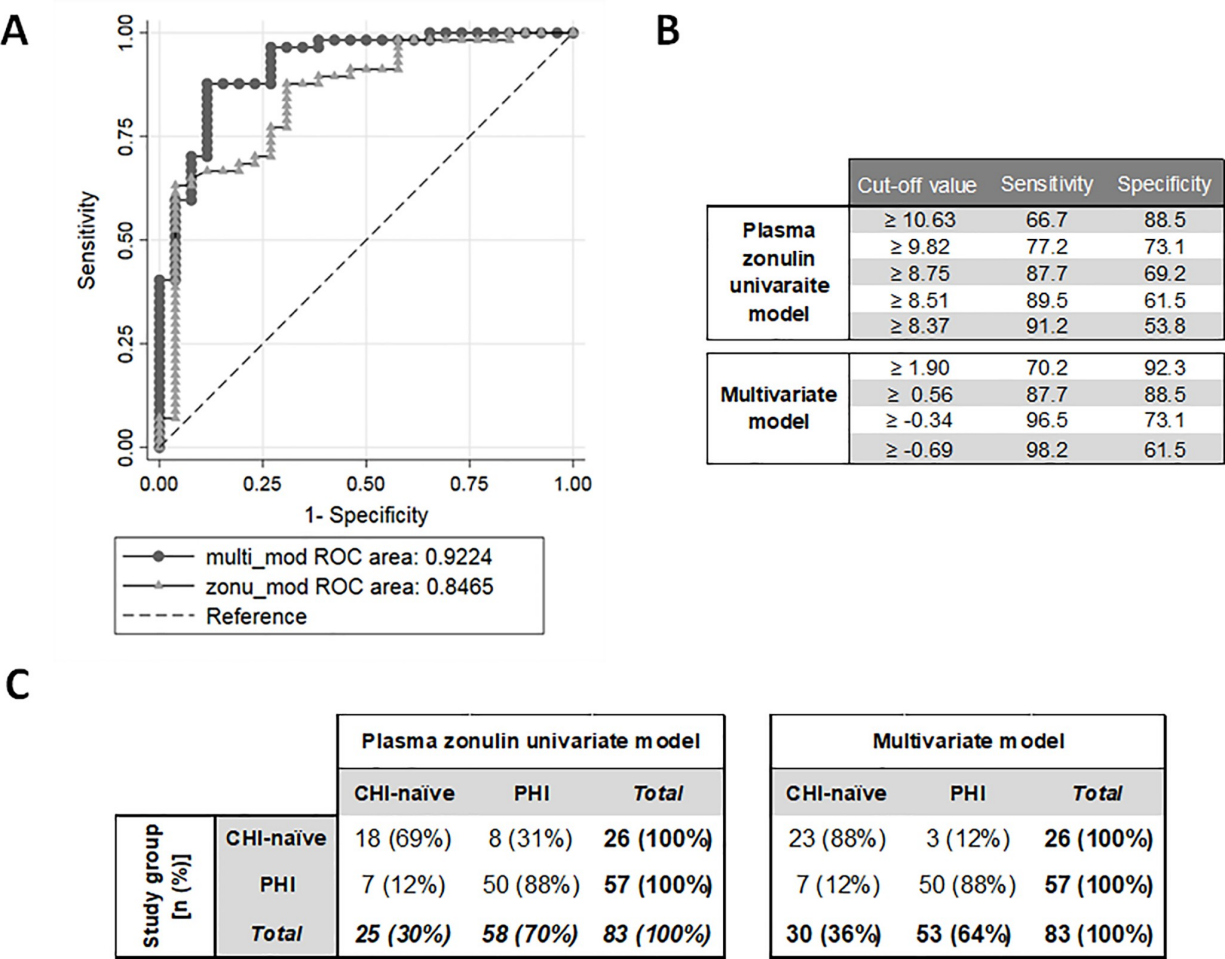
Performance of univariate and multivariate biomarkers models in predicting primary HIV infection. A) Comparison between ROC curves for plasma zonulin univariate model and multivariate model adjusted by age, plasma zonulin and plasma sCD14. AUC as area under the curve. B) Cut-off values for plasma zonulin univariate model (ng/mL) and multivariate model (score) with their respective sensitivity and specificity values. C) Prediction of plasma zonulin univariate model with a cutoff of ≥ 8.75 ng/mL was compared to that of the multivariate model with a score cutoff of ≥ 0.56.

An adjusted multivariate model including age, plasma zonulin and sCD14 according to the formula [*score = -10*.*81–0*.*08 age + 10*.*28 log10 (zonulin) + 10*.*70 log10(sCD14)]* further increased the classification performance (AUC = 0.92 [95% CI 0.86–0.99]; Fig 4A). For the univariate zonulin model, a cutoff value of plasma zonulin ≥ 8.75 ng/mL was able to identify PHI with 87.7% sensitivity (95% CI, 76.3–94.9) and 69.2% specificity (95% CI, 48.2–85.7; Fig 4B). In the multivariate model, a cut-off score of ≥ 0.56 provided the same sensitivity of 87.7% (95% CI, 76.3–94.9) but a higher specificity of 88.5% (95%CI 69.8–97.6; Fig 4B). Thus both models correctly identified 50/57 PHI-individuals but differed in their identification of CHI-naïve individuals. The zonulin-based univariate and multivariate models correctly classified 18/26 and 23/26 CHI-naïve individuals, respectively (Fig 4C). Consequently, univariate and multivariate classification methods showed a substantial level of agreement for PHI detection with kappa-statistic of 0.67 (95%CI 0.50–0.84).

## Discussion

In this study we described the dynamics of plasma and stool GI-damage biomarkers and their association with HIV-disease markers during the first year of HIV-infection. We found that higher plasma sCD14 was significantly associated with increased VL and decreased CD4/CD8 ratio during PHI, whereas lower stool HBD2 was significantly associated with self-reported intestinal complaints. In addition, assessing these biomarkers as indicators of HIV-recency, plasma zonulin demonstrated a good classification performance identifying 87.7% of the PHI and 69.2% of the CHI-individuals in our cohort.

Among the GI-damage biomarkers assessed, plasma sCD14 showed the most significant increase in the early stages of HIV-infection as compared to HIV-uninfected individuals. We additionally found that higher plasma sCD14 levels during the first year of infection were associated with higher viremia levels and lower CD4/CD8 ratios, immunological variables that at set point have been previously reported to be surrogates of disease progression and mortality [42]. Indeed, sCD14 is a marker of acute phase response and/or monocyte activation and previous studies have reported that higher plasma sCD14 levels during PHI predict rapid HIV-progression [43,44]. However, this biomarker also binds to lipopolysaccharides in plasma [45] and thus has been employed as a surrogate of microbial translocation. Several studies have shown that plasma levels of sCD14 are involved in the immunopathogenesis of HIV infection [46] and are an independent predictor of morbidity [47] and mortality [30,48] among CHI-patients. Plasma EndoCab IgM, a surrogate of lipopolysaccharide, was also associated with PHI but performed more poorly than sCD14 in predicting PHI, suggesting that both viral-induced monocyte activation, reflected by sCD14, and microbial translocation, reflected by Endocab IgM, may occur at early stages. However, we were unable to determine whether sCD14 association with markers of HIV-disease was solely a consequence of early innate immune activation or an indicator of microbial translocation. Measurements of the sCD14 bacterial 16S DNA and plasma lipopolysaccharides kinetics during the different stages of HIV infection would be useful to shed light on the crucial processes that characterize HIV-inflicted immune and GI damage.

The stool biomarkers described in this manuscript were those generally used in the diagnosis and management of IBD-patients [18,19]. Calprotectin and lactoferrin, both markers of neutrophil activation, are the most commonly used fecal markers for intestinal inflammation in IBD [21,49]. In our study, stool lactoferrin was significantly increased in patients at 2 months post-HIV-infection as compared to HIV-uninfected individuals and normalized in the CHI-individuals. This increase was also observed for stool calprotectin, however, in this case, levels did not normalize in the CHI. Since intestinal epithelial-damage is irreversible in untreated HIV-infected individuals [5,6], elevated calprotectin levels during PHI and CHI may be a consequence of the continued GI-damage. Indeed, a previous study in HIV-infected ART-naive individuals found elevated fecal calprotectin levels in half of the patients regardless of stage [11]. On the other hand, decreased level of stool lactoferrin in CHI individuals suggests that its presence in stool during PHI may more closely reflect acute inflammation rather than advanced intestinal-damage. In order to shed light on the role and timing of these GI-damage biomarkers, future studies should asses their expression in plasma and stool along with gut biopsies at different stages of HIV infection.

Despite the association described between stool biomarkers and disease flares in IBD-patients [18,19], no significant associations were found between stool IBD-biomarker and markers of HIV-disease such as VL or CD4/CD8 ratio during PHI. We did observe an association between lower stool HBD2 levels during the first year of HIV infection and self-reported intestinal complaints. HBD2 is an antimicrobial peptide secreted by diverse epithelial cells, including intestinal epithelial cells, after an infection or during inflammatory process in chronic diseases such as IBD [50,51]. The massive GALT destruction produced during PHI [7,8] leads to increased apoptosis in the GI epithelial cells producers of HBD2 [52]. HBD2 levels tended to be higher in both CHI and PHI during the first year post-infection as compared to HIV-uninfected individuals. Thus, the etiology of the association between lower HBD2 levels and self-reported intestinal complaints in PHI individuals is likely to be multifactorial and may be affected by the presence of other Gl infections or the massive inflammatory response triggered after HIV-infection.

Although some stool IBD-biomarkers were differentially expressed between PHI and CHI, they did not provide sufficient predictive ability to identify PHI. Plasma zonulin demonstrated the best accuracy to distinguish PHI from CHI-naïve individuals. Since some biomarker levels have been previously described to be affected by age [53,54], gender [55] or VL [23,25,34], these covariates were also considered in the construction of a multivariate model. The resulting multivariate model retained plasma zonulin and sCD14 together with age. The significantly higher age in the CHI-naïve individuals in our cohort as compared to the PHI likely explains age as a covariate [33]. This multivariate model slightly increased the model specificity but showed a substantial agreement in the classification performance with respect to the univariate model. These models showed a moderate accuracy and high uncertainty in the performance of plasma zonulin to distinguish PHI from CHI, thus hampering its use as a simple HIV incidence assay as compared to existing robust serological tests [56,57]. Further studies combining plasma zonulin with additional inflammatory biomarkers or HIV-specific antibody subtypes might improve the model performance for PHI identification and HIV-incidence estimations. Zonulin is a protein expressed by viable gut epithelial cells that modulates the permeability of tight junctions between cells of the wall of the digestive tract and its release has been documented to be triggered by small intestinal exposure to bacteria and gliadin [58]. It is uncertain why plasma zonulin levels were significantly lower in CHI-individuals, but greater gut epithelial cell death or malfunction during AIDS might decrease its expression. These findings are in line with previous studies that reported a strong association between plasma zonulin levels and mortality in HIV-infected individuals, even after adjustment for proximal CD4 T-cell count [48].

As a limitation from the study design, data for the stool biomarkers was not available at the first month of infection. This would have allowed a further characterization of the earliest responses at the GI-level simultaneous to the massive CD4-T cell destruction in the GALT [7,8]. Similarly, the loss to follow-up along the longitudinal visits may have resulted in insufficient power to detect associations. Additionally, the high burden of GI-infections prevalent in the study area could have impacted the biomarker dynamics and expression levels. We did not observe any significant difference in the biomarker levels according to the GI-infection status in the HIV-infected individuals. However, due to sample size restrictions, the presence of blood in stool, the detection of a GI protozoa or a bacterial infection were equally considered as positive results for GI-infection [33]. Future studies should evaluate the specific effect that different GI-infections could have on the dynamics of these biomarkers during HIV infection.

In conclusion, our results do not support the hypothesis that stool IBD-biomarkers can be used as surrogates of HIV-disease progression or to identify PHI among HIV-infected individuals. However, we found that higher plasma sCD14 was significantly associated with increased viremia levels and more compromised immune system during PHI. Our results also point to stool lactoferrin, calprotectin and zonulin as potential indicators of different GI pathogenic events following GALT destruction and inflammation during PHI.

## Supporting information

S1 Table Biomarker concentration by study group. (DOCX)

S2 Table Significance level of median biomarker comparison between study groups. (DOCX)
