# Supplementary material for: Different pattern of stool and plasma gastrointestinal damage biomarkers during primary and chronic HIV infection
Source: PLoS One. 2019 Jun 11;14(6):e0218000. doi: 10.1371/journal.pone.0218000 (PMC6559643; doi:10.1371/journal.pone.0218000)
Supplement: S1 Table — (DOCX) [file pone.0218000.s001.docx]

**Supporting information**

|  | **PHI at M2** | | | | |  | **HIV-uninfected** | | | | |  | **CHI-naive** | | | | |  | **CHI-ART** | | | | |
| --- | --- | --- | --- | --- | --- | --- | --- | --- | --- | --- | --- | --- | --- | --- | --- | --- | --- | --- | --- | --- | --- | --- | --- |
| BIOMARKER | N | Median | Q1 | Q3 | % Det. |  | N | Median | Q1 | Q3 | % Det. |  | N | Median | Q1 | Q3 | % Det. |  | N | Median | Q1 | Q3 | % Det. |
| FABP2 (pg/mL) | 29 | 1074.03 | 651.25 | 1461.63 | 100 |  | 58 | 821.92 | 595.77 | 1070.67 | 100 |  | 26 | 1132.12 | 859.91 | 1352.97 | 96.15 |  | 30 | 1106.39 | 878.69 | 1400.49 | 100 |
| LBP (µg/mL) | 29 | 9.77 | 6.74 | 11.58 | 96.55 |  | 58 | 6.23 | 4.47 | 8.13 | 96.55 |  | 26 | 7.87 | 4.88 | 10.82 | 96.15 |  | 30 | 5.84 | 4.23 | 8.25 | 100 |
| sCD14 (µg/mL) | 29 | 2.38 | 2.04 | 2.71 | 100 |  | 58 | 1.82 | 1.54 | 2.09 | 100 |  | 26 | 2.21 | 1.96 | 2.43 | 100 |  | 30 | 2.82 | 2.33 | 3.4 | 100 |
| zonulin (ng/mL) | 29 | 11.74 | 8.92 | 14.07 | 100 |  | 58 | 12.17 | 9 | 14.27 | 100 |  | 26 | 8.14 | 6.52 | 9.98 | 92.31 |  | 30 | 6.41 | 1.75 | 8.7 | 80 |
| EndoCab IgG (GMU) | 29 | 132.64 | 82.57 | 182.83 | 100 |  | 58 | 153.92 | 81.45 | 333.63 | 98.28 |  | 26 | 145.56 | 70.49 | 238.08 | 100 |  | 30 | 121.45 | 98.39 | 255.89 | 96.67 |
| EndoCab IgM (GMU) | 29 | 92.89 | 63.91 | 154.52 | 100 |  | 58 | 116.16 | 74.77 | 158.34 | 100 |  | 26 | 84.65 | 55.21 | 107.09 | 100 |  | 30 | 50.3 | 34.88 | 98.91 | 100 |
| IgA ASCA (AU) | 29 | 1.95 | 1.07 | 2.84 | 89.66 |  | 58 | 1.07 | 0.66 | 2.43 | 89.66 |  | 26 | 0.85 | 0.58 | 1.41 | 76.92 |  | 30 | 1.03 | 0.65 | 2.77 | 90 |
| IgGASCA (AU) | 29 | 3.56 | 2.35 | 5.64 | 100 |  | 58 | 2.11 | 1.04 | 3.32 | 100 |  | 26 | 2.09 | 0.94 | 3.82 | 96.15 |  | 30 | 2.87 | 1.34 | 8.4 | 96.67 |
| *VEGF (pg/mL)* | 29 | 0.09 | 0.09 | 0.09 | 24.14 |  | 58 | 0.09 | 0.09 | 0.09 | 20.69 |  | 26 | 0.09 | 0.09 | 3.11 | 38.46 |  | 30 | 0.09 | 0.09 | 0.09 | 23.33 |
| *IL-17 (pg/mL)* | 29 | 0.33 | 0.33 | 0.33 | 10.34 |  | 58 | 0.33 | 0.33 | 0.33 | 17.24 |  | 26 | 0.33 | 0.33 | 3.67 | 34.62 |  | 30 | 0.33 | 0.33 | 0.33 | 13.33 |
| EDNEPX (g/g) | 20 | 0.71 | 0.51 | 1.32 | 100 |  | 49 | 0.42 | 0.32 | 0.73 | 100 |  | 21 | 0.54 | 0.31 | 0.95 | 100 |  | 25 | 0.23 | 0.16 | 0.4 | 100 |
| PMNElastase (g/g) | 20 | 0.14 | 0.08 | 0.19 | 100 |  | 49 | 0.11 | 0.05 | 0.17 | 100 |  | 20 | 0.17 | 0.09 | 0.22 | 100 |  | 25 | 0.14 | 0.12 | 0.21 | 100 |
| Lactoferrin (g/g) | 20 | 5.22 | 0.61 | 10 | 100 |  | 49 | 0.64 | 0.14 | 1.37 | 100 |  | 21 | 0.33 | 0.18 | 1.46 | 100 |  | 25 | 0.31 | 0.11 | 0.85 | 100 |
| HBD2 (ng/g) | 20 | 63.25 | 50.82 | 78.39 | 100 |  | 49 | 46.33 | 36.95 | 70.45 | 100 |  | 21 | 65.33 | 33.67 | 104.41 | 100 |  | 25 | 65.89 | 30.96 | 96.47 | 100 |
| sIgA (mg/g) | 20 | 1.19 | 1.02 | 1.45 | 100 |  | 49 | 1.08 | 0.89 | 1.55 | 100 |  | 21 | 1.18 | 0.5 | 2.01 | 100 |  | 25 | 1.14 | 0.91 | 1.74 | 96 |
| S100A12 (g/g) | 20 | 13.35 | 6.77 | 28.25 | 95 |  | 49 | 6.26 | 3.24 | 16.1 | 95.92 |  | 20 | 7.07 | 4.16 | 15.2 | 90 |  | 25 | 6.41 | 3.94 | 13.7 | 96 |
| α1-Antitrypsin (mg/g) | 20 | 0.11 | 0.07 | 0.18 | 100 |  | 49 | 0.07 | 0.05 | 0.11 | 97.96 |  | 21 | 0.11 | 0.09 | 0.13 | 100 |  | 25 | 0.1 | 0.08 | 0.14 | 100 |
| Zonulin (ng/g) | 20 | 26.06 | 21.75 | 35.42 | 100 |  | 49 | 24.88 | 18.35 | 29.91 | 100 |  | 21 | 47.83 | 26.1 | 57 | 100 |  | 25 | 44.5 | 30.18 | 54.86 | 100 |
| pANCA (OD) | 20 | 0.05 | 0.04 | 0.07 | 100 |  | 49 | 0.06 | 0.04 | 0.09 | 100 |  | 20 | 0.05 | 0.03 | 0.08 | 100 |  | 25 | 0.04 | 0.03 | 0.22 | 100 |
| ASCA (OD) | 20 | 0.01 | 0 | 0.01 | 100 |  | 49 | 0.01 | 0 | 0.01 | 100 |  | 20 | 0 | 0 | 0.01 | 100 |  | 25 | 0.01 | 0 | 0.01 | 100 |
| *Calprotectin (g/g)* | 20 | 19.04 | 5.94 | 33.78 | 95 |  | 49 | 0.01 | 0.01 | 7.69 | 44.9 |  | 20 | 9.72 | 6.21 | 47.84 | 95 |  | 25 | 9.27 | 4.44 | 18.97 | 100 |
| *Claudin3 (ng/g)* | 20 | 0.07 | 0.05 | 0.13 | 100 |  | 49 | 0.08 | 0.05 | 0.17 | 100 |  | 20 | 0.06 | 0.04 | 0.13 | 100 |  | 25 | 0.1 | 0.06 | 0.17 | 100 |

**S1 Table. Biomarker concentration by study group.**

**S1 Table footnotes**: PHI, primary HIV infection; M, months after HIV infection; CHI, chronic HIV infection; CHI-ART, CHI on antiretroviral-treatment; Q, quartile; % Det., % quantifiable samples. Units measured per gram refer to gram of stool.
