# Supplementary material for: Different pattern of stool and plasma gastrointestinal damage biomarkers during primary and chronic HIV infection
Source: PLoS One. 2019 Jun 11;14(6):e0218000. doi: 10.1371/journal.pone.0218000 (PMC6559643; doi:10.1371/journal.pone.0218000)
Supplement: S2 Table — (DOCX) [file pone.0218000.s002.docx]

**Supporting information**

| **Biomarker** | **Group comparison** | **PHI at M2 vs. Ctrl** | **CHI-naive vs. Ctrl** | **CHI-ART vs. Ctrl** | **CHI-naive vs. PHI at M2** | **CHI-ART vs. PHI at M2** | **CHI-naive vs. CHI-ART** |
| --- | --- | --- | --- | --- | --- | --- | --- |
| sCD14 (µg/mL) | < 0,0001 | < 0,0001 | 0,0083 | < 0,0001 | 0,6996 | 0,3144 | 0,0314 |
| *Calprotectin (g/g)*** | < 0,0001 | < 0,0001 | 0,0001 | < 0,0001 | 0,9244 | 0,7415 | 0,9244 |
| LBP (µg/mL) | 0,0023 | 0,0025 | 0,2566 | 0,9967 | 0,5400 | 0,0208 | 0,4748 |
| Lactoferrin (g/g) | 0,0053 | 0,0149 | 0,9884 | 0,8855 | 0,0257 | 0,0066 | 0,9886 |
| IgGASCA (AU) | 0,0171 | 0,0159 | 0,9994 | 0,4346 | 0,0797 | 0,5798 | 0,6527 |
| α1-Antitrypsin (mg/g) | 0,0239 | 0,1703 | 0,0703 | 0,1143 | 0,9911 | 1,0000 | 0,9913 |
| FABP2 (pg/mL) | 0,0216 | 0,2449 | 0,0457 | 0,0940 | 0,8851 | 0,9787 | 0,9857 |
| S100A12 (g/g) | 0,3180 | 0,3435 | 0,9998 | 0,9925 | 0,5438 | 0,3245 | 0,9909 |
| IgA ASCA (AU) | 0,1614 | 0,3882 | 0,7444 | 1,0000 | 0,1185 | 0,5127 | 0,8108 |
| HBD2 (ng/g) | 0,4360 | 0,4354 | 0,8322 | 0,7121 | 0,9409 | 0,9679 | 0,9991 |
| EDNEPX (g/g) | 0,0004 | 0,4418 | 0,9851 | 0,0065 | 0,7642 | 0,0004 | 0,0143 |
| PMNElastase (g/g) | 0,0801 | 0,5281 | 0,1993 | 0,1320 | 0,9551 | 0,9385 | 1,0000 |
| EndoCab IgG (GMU) | 0,7295 | 0,7368 | 0,9937 | 0,8604 | 0,9235 | 0,9970 | 0,9733 |
| EndoCab IgM (GMU) | 0,0003 | 0,8055 | 0,0953 | 0,0002 | 0,5744 | 0,0260 | 0,4868 |
| Claudin3 (ng/g) | 0,3087 | 0,9010 | 0,6393 | 0,8200 | 0,9765 | 0,5478 | 0,2942 |
| sIgA (mg/g) | 0,8288 | 0,9020 | 0,9995 | 0,9171 | 0,9059 | 0,9998 | 0,9210 |
| Zonulin (ng/g) | 0,0004 | 0,9652 | 0,0046 | 0,0052 | 0,0786 | 0,1002 | 0,9970 |
| zonulin (ng/mL) | < 0,0001 | 0,9692 | 0,0004 | < 0,0001 | 0,0006 | < 0,0001 | 0,5634 |
|  | | | |  |  |  |  |

**S2 Table. Significance level of median biomarker comparison between study groups.**

**S2 Table footnotes:** Individual and group comparisons were performed using Nemenyiand Kruskal-Wallis test, respectively. Individual and group comparisons of left-censored data (**) were performed using Peto-Peto test with Holm adjust for multiple comparisons. M, months after HIV infection; Ctrl, HIV-uninfected; PHI, primary HIV infection; CHI, chronic HIV infection; CHI-ART, CHI on antiretroviral-treatment. Units measured per gram refer to gram of stool.
